# Systems Biology Investigation of cAMP Modulation to Increase SMN Levels for the Treatment of Spinal Muscular Atrophy

**DOI:** 10.1371/journal.pone.0115473

**Published:** 2014-12-16

**Authors:** Sean G. Mack, Daniel J. Cook, Prasad Dhurjati, Matthew E. R. Butchbach

**Affiliations:** 1 Center for Applied Clinical Genomics, Nemours Biomedical Research, Nemours Alfred I. duPont Hospital for Children, Wilmington, Delaware, United States of America; 2 Center for Pediatric Research, Nemours Biomedical Research, Nemours Alfred I. duPont Hospital for Children, Wilmington, Delaware, United States of America; 3 Department of Chemical and Biomolecular Engineering, University of Delaware, Newark, Delaware, United States of America; 4 Department of Biological Sciences, University of Delaware, Newark, Delaware, United States of America; 5 Department of Pediatrics, Thomas Jefferson University, Philadelphia, Pennsylvania, United States of America; University of Edinburgh, United Kingdom

## Abstract

Spinal muscular atrophy (SMA), a leading genetic cause of infant death worldwide, is an autosomal recessive disorder caused by the loss of *SMN1 (survival motor neuron 1)*, which encodes the protein SMN. The loss of *SMN1* causes a deficiency in SMN protein levels leading to motor neuron cell death in the anterior horn of the spinal cord. *SMN2*, however, can also produce some functional SMN to partially compensate for loss of *SMN1* in SMA suggesting increasing transcription of *SMN2* as a potential therapy to treat patients with SMA. A cAMP response element was identified on the *SMN2* promoter, implicating cAMP activation as a step in the transcription of *SMN2*. Therefore, we investigated the effects of modulating the cAMP signaling cascade on SMN production *in vitro* and *in silico*. SMA patient fibroblasts were treated with the cAMP signaling modulators rolipram, salbutamol, dbcAMP, epinephrine and forskolin. All of the modulators tested were able to increase gem formation, a marker for SMN protein in the nucleus, in a dose-dependent manner. We then derived two possible mathematical models simulating the regulation of *SMN2* expression by cAMP signaling. Both models fit well with our experimental data. *In silico* treatment of SMA fibroblasts simultaneously with two different cAMP modulators resulted in an additive increase in gem formation. This study shows how a systems biology approach can be used to develop potential therapeutic targets for treating SMA.

## Introduction

Spinal muscular atrophy (SMA) is an autosomal recessive, neurodegenerative disorder characterized by the progressive loss of α-motor neurons in the anterior horn of the spinal cord; this loss leads to progressive muscle weakness and atrophy [1]. SMA is a leading genetic cause of infant death worldwide with 1 in 5000–10,000 children born with the disease [2], [3]. Loss of or mutation in *SMN1* (*survival motor neuron 1*) leads to SMA [4]. In humans and only in humans, *SMN1* is duplicated to yield *SMN2* [5], [6]. There is a single nucleotide change (C→T) within *SMN2* exon 7 that causes most of *SMN2* mRNAs to lack exon 7 (SMNΔ7). The resultant SMNΔ7 protein is unstable and not fully functional [7], [8]. *SMN2* can, however, provide some full-length, functional SMN (FL-SMN) protein. The number of *SMN2* copies modifies disease severity in SMA patients [9]–[16]. In transgenic mouse models for SMA, the copy number of human *SMN2* modulates the phenotypic severity [17]–[19]. *SMN2* is, therefore, an endogenous genetic modifier of disease severity in SMA.

**Figure 1:**
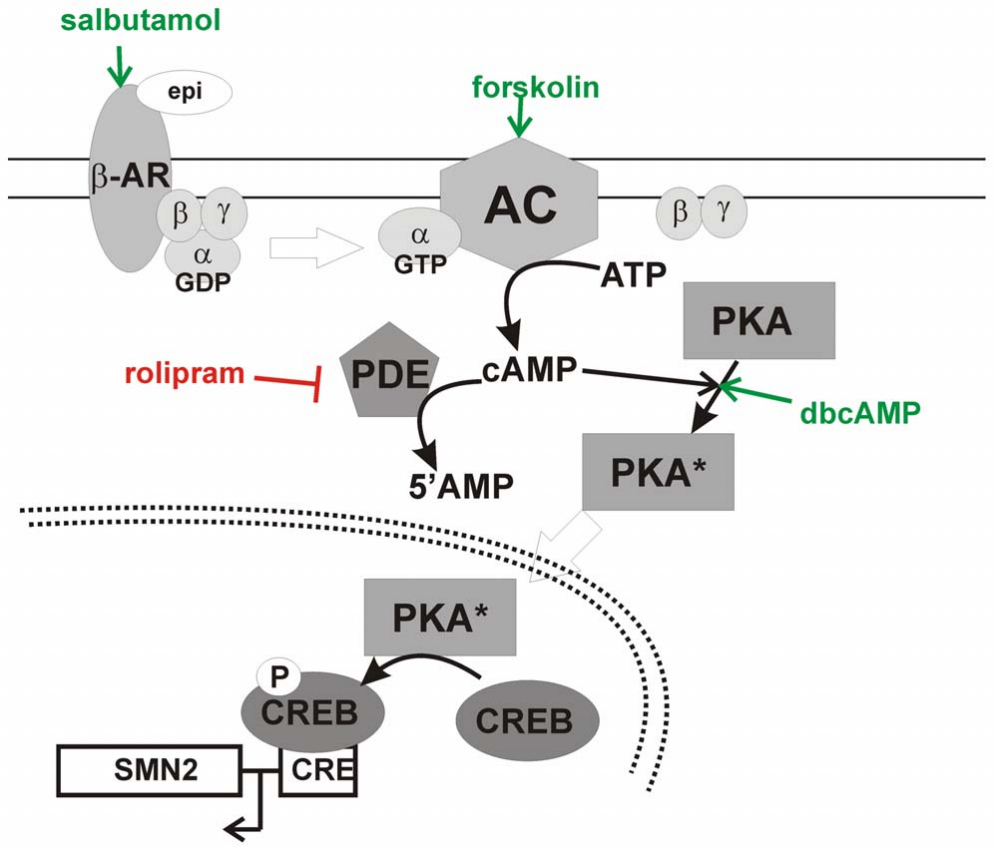
The cAMP pathway and *SMN2* expression. In this signaling cascade, ligand activation of the membrane bound G protein-coupled receptors such as the β-adrenergic receptor (βAR) results in the dissociation of the G_α,s_ subunit from the receptor. G_α,s_ then activates adenylyl cyclase (AC). Once stimulated, AC produces cAMP by cyclizing intracellular ATP. cAMP then activates a serine/threonine kinase known as cAMP-dependent protein kinase—or protein kinase A (PKA). The regulatory subunits of PKA are then released and the catalytic subunit acts on many downstream targets including the cAMP-response element-binding (CREB) protein. Phosphorylated CREB (phospho-CREB) binds to cAMP response elements (CREs) with the promoter regions of various genes including *SMN2*. cAMP signaling is attenuated by cyclic nucleotide phosphodiesterases (PDEs) which break down cAMP into AMP.

Because of this phenotype modifying property, *SMN2* has been the target for numerous drug discovery strategies. Targeting cyclic adenosine monophosphate (cAMP) signaling is of particular interest in developing inducers of *SMN2* expression. The cAMP signaling cascade (**Fig. 1**) is used by both prokaryotes and eukaryotes to regulate various processes including cell growth, metabolism and stress response [20]. The *SMN2* promoter contains at least one cAMP-response element (CRE) that is able to bind to activated CRE-binding protein (phospho-CREB) [21]. The β2-adrenergic agonist salbutamol increases the amount of FL-SMN protein in SMA fibroblasts and leukocytes of SMA patients [22], [23]. Forskolin, which stimulates adenylyl cyclase (AC) catalysis to produce cAMP from ATP, increases *SMN2* promoter activity [21]. The synthetic analogue dibutyryl cAMP (dbcAMP)—which activates cyclic AMP-dependent protein kinase (PKA)—also increases *SMN2* promoter activity [21]. Taken together, these studies show that modulation of cAMP signaling can increase SMN expression from *SMN2*.

In this study, we will examine the effect of modulating cAMP signaling on SMN expression in SMA cells using a systems biology approach. cAMP signaling will be regulated by one of four possible targets (**Fig. 1**): activation of G protein-coupled receptors, activation of AC, activation of PKA and inhibition of phosphodiesterases (PDEs) that break down cAMP. Within the nuclei of most eukaryotic cells, SMN protein localizes to discreet foci known as gems, or gemini of coiled bodies [24]. The number of gems are markedly reduced within the nuclei of cells from SMA patients [9]. Using gem formation as an indicator of the expression of functional, FL-SMN protein, we will develop computational models of interactions between the cAMP pathway and gem formation to investigate how modulating cAMP signaling dynamics affects FL-SMN production. This systems biology approach consists of a synergistic interaction between experimental data and mathematical models. The experimental data and domain knowledge are used to develop the initial models and through a process of iteration, the model assumptions are updated to come up with an improved model [25]. Such a systems modeling approach can ultimately aid in the development and optimization of cAMP signaling-based therapeutic strategies for SMA.

## Materials and Methods

### Fibroblast Cell Culture

GM03813 fibroblasts [26] (Coriell Cell Repositories; Camden, NJ) were derived from a SMA patient with deletion of *SMN1* and 2 copies of *SMN2*. GM03814 fibroblasts [26] (Coriell Cell Repositories) were derived from the carrier mother of GM03813; this line, therefore, carries 1 copy of *SMN1*. All fibroblast lines were maintained in Dulbecco's modified essential medium (DMEM; Life Technologies, Grand Island, NY) containing 10% fetal bovine serum (FBS; Atlas Biologicals, Fort Collins, CO), 2 mM L-glutamine (Life Technologies) and 1% penicillin/streptomycin (Life Technologies).

### Drug Treatment

Cells were seeded onto gelatinized glass coverslips at a density of 4000 cells/cm^2^. Cells were treated with one of the following compounds (n = 3/dose): dbcAMP (5–500 µM; EMD Millipore, Billerica, MA), epinephrine (1–100 nM; Sigma-Aldrich, St. Louis, MO), forskolin (0.5–50 µM; EMD Millipore), salbutamol (1–100 nM; Sigma-Aldrich), rolipram (0.1–10 µM; Sigma-Aldrich) or vehicle. ddH_2_O was utilized as the vehicle for the dbcAMP treatments while DMSO was the vehicle for the remaining compounds. Test compounds were added to the medium at a 1∶1000 dilution. Medium was changed daily and fresh compound was added for 5 days.

### Immunofluorescence

Immunostaining of fibroblast cells was accomplished as described previously [27]. Briefly, cells grown on gelatinized coverslips were fixed with Fixative Buffer (2% paraformaldehyde, 400 µM CaCl_2_, 50 mM sucrose in 100 mM sodium phosphate buffer, pH 7.4) for 30 min at room temperature, thoroughly rinsed with phosphate-buffered saline (PBS) and permeabilized with ice-cold acetone for 10 min. After drying for at least 30 min at room temperature, the cells were rehydrated with PBS for 10 min at room temperature and then blocked with 5×BLOCK for 60 min at room temperature. The cells were incubated overnight with primary antibody solution (mouse anti-SMN mAb (MANSMA2 (8F7); Developmental Studies Hybridoma Bank, Iowa City, IA [28]) diluted 1∶200 in 1×BLOCK) at 4°C. The cells were then washed extensively (3×10 min) with PBS and incubated with secondary antibody solution (biotinylated goat anti-mouse IgG (Jackson ImmunoResearch) diluted 1∶400 with 1×BLOCK) for 60 min at room temperature. The cells were then washed with PBS (3×10 min) and then incubated with AlexaFluor 594-conjugated streptavidin (Life Technologies) diluted 1∶200 with PBS for 60 min at room temperature. Cells were then counterstained with Hoescht 33342 (1 µg/mL; Life Technologies) in PBS for 5 min. After thorough washing with PBS, coverslips were mounted onto glass slides with ImmuMount (Shandon Lipshaw) and stored at 4°C until analysis.

### Gem Count Analysis

SMN immunostaining in fibroblasts was visualized using a DMRXA2 epifluorescence microscope (Leica Microsystems) with an ORCA-ER cooled camera (Hamamatsu, Hamamatsu City, Japan) and Volocity 6.1.1 software (Perkin-Elmer). For gem counting, the following parameters were measured in 100 randomly selected nuclei: the number of gems, the number of cells with gems and the number of cells with more than 1 gem. The gem counts were converted into a concentration value (in mM) using an approximate average cell volume (2.68×10^−13^ L). This conversion assumes that all cells are of equal volume [29].

### Development of Mathematical Models

The computational model was visualized and developed in CellDesigner [30]. Each model was then converted into Systems Biology Markup Language (SBML) format [31] and imported into MATLAB using SBToolbox [32]. SBToolbox was used to complete parameter sensitivity and model simulations.

### Simplified cAMP Model

#### Model Derivation

In order to adapt the Williamson model [33] to our system, their parameters required reworking to be in accordance with the units of our data. The Williamson model was shown to match data from literature [34], [35] which is reported in units of nmol per gram wet weight versus minutes. Through careful inspection, the time units of the model were determined to be 20 seconds (s) (i.e. one time unit represents 20 s in the data) with the concentration units remaining the same as the data. The data points of the model results presented in Williamson were extracted using WebPlotDigitizer v.2.6 (http://arohatgi.info/WebPlotDigitizer) and converted into the proper units.

Our simplified model consists of three ordinary differential equations (ODEs; **Equations 1** **–** **3**) and two conservation equations (**Equations 4** **&** **5**). **Equation 1** represents the rate of change in the active form of the G protein receptor (GP_a_) in the presence or absence of a stimulatory hormone or agonist. **Equation 2** models the instantaneous changes in active PKA (PKA_a_) as a result of cAMP production. **Equation 3** captures both cAMP production from ATP by AC and degradation into AMP via PKA_a_-stimulated PDE activity. The conservation equations demonstrate the balance between the active and inactive forms of both GP and PKA.

In order to further simplify the system, the two PDE terms from the Williamson *et al*. model [33] were combined into one term. As evinced in the Williamson *et al*. study [33], PDE2 does not have a significant effect on cAMP concentration, especially when compared to PDE1. Since there are multiple isoforms of PDEs in humans [36], we generated a general PDE term to model the overall inhibitory action of PDEs in the cells. The model parameters were defined as in [33] and their descriptions are summarized in **Table 1**.

**Table 1:** Optimized parameter values for cAMP pathway full cAMP:*SMN2* model.

Parameter	Description	Value	Units	Source
GP k_f_	G-Protein Activation Rate	1.33×10^−7^	(mMmin)^−1^	This Work
GP k_r_	G-Protein Deactivation Rate	2.05×10^−7^	min^−1^	This Work*
PKA k_f_	PKA Activation Rate	5.37	(mMmin)^−1^	This Work*
PKA k_r_	PKA Deactivation Rate	0.65	min^−1^	This Work*
AC_basal_	Base AC Activation Rate	1.00×10^−5^	mM/min	This Work*
GP k_a_	G-Protein Catalysis Rate	87.83	min^−1^	This Work*
PKA K_i_	PKA Inhibition Rate	1419.09	mM^−1^	This Work*
V_max_PDE	Max cAMP Degradation Rate	1.75	min^−1^	This Work
K_m_PDE	PDE Dissociation Rate Constant	7.79	mM	This Work
CREB k_f_	CREB Phosphorylation Rate	59.54	(mMmin)^−1^	This Work
CREB k_r_	CREB De-phosphorylation Rate	0.93	min^−1^	This Work
k_max_	Max Transcription Rate	1.14×10^−5^	mM/min	This Work
c	Promoter Binding Efficiency	8.61×10^−3^	mM^−1^	This Work
d_m_	mRNA Degradation Rate Constant	2.10×10^−3^	min^−1^	[56]
H	Hill Coefficient	1	n/a	This Work
k_p_	Translation Rate Constant	9109.61	min^−1^	This Work
d_p_	FL-SMN Degradation Rate Constant	2.70×10^−3^	min^−1^	[39]
k_g_	Gem Formation Rate Constant	3.07×10^−6^	min^−1^	This Work
d_g_	Gem Degradation Rate Constant	7.70×10^−4^	min^−1^	[39]
Fk_a_	Forskolin Catalysis Rate Constant	0.027	min^−1^	This Work
α	Salbutamol Splicing Constant	7172.40	mM^−1^	This Work
K_I_	Rolipram Inhibition Constant	1.71×10^−5^	mM	This Work

The parameters marked with an asterisk (*) were carried over from the simplified cAMP model.



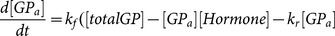
(1)

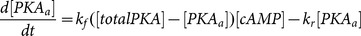
(2)

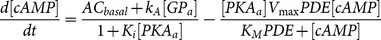
(3)

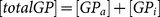
(4)

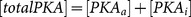
(5)


The model was then fit to the converted experimental data using the optimization algorithms detailed below. The optimized parameters determined from this model fitting are listed in **Table 1**.

The experimental data utilized by Williamson et al. [33] represented concentration in units of nmol per gram wet weight (gww). However, the glucose pulse used in this study was in units of mM. In order to convert the data into mM concentrations, the following equation was used:
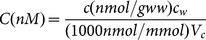
(6)where C(nM) is concentration of cAMP in milimolar, C_w_ is the conversion factor to grams dry weight (0.15) and V_c_ is the volume of 10^7^ cells (2.68×10^−6^ L; approximately 10^7^ cells in 1 gww). The equation and constant values were adapted from the equation detailed in Williamson et al. [33].

#### Parameter estimation

Parameters for the base model were estimated by fitting our modified model to the data extracted from the Williamson results through a simulated annealing algorithm [37], [38], which is efficient at finding the global minimum for optimization. The algorithms were taken from Systems Biology Toolbox (SBToolbox) [32] in MATLAB and the estimation was run using the SBparameterestimation function from SBToolbox as well as the main estimation functions listed in additional files.

### Full cAMP:*SMN2* Model

#### Model Development

In order to capture the treatment pathway of interest, the downstream effectors of the base model were incorporated to develop a model of how the cAMP pathway affects FL-SMN protein production and gem formation. Our full cAMP pathway model encompasses the base model equations with four additional ODEs (**Equations 7** **–** **10**) and one additional conservation equation (**Equation 11**). ODEs were derived for CREB phosphorylation by PKA_a_ (**Equation 7**), *SMN2* promotion by phospho-CREB (**Equation 8**), translation of *FL-SMN* mRNA into FL-SMN protein (**Equation 9**) and the self-assembly of FL-SMN into gems (**Equation 10**). *SMN2* activation was modeled using the Hill equation while the rest of the parameters were based on mass action kinetics. The conservation relationship detailed in **Equation 11** represents the total CREB molecules in the cell. Each model parameter is defined in **Table 1**. Our model used units of min for time and mM for concentration.
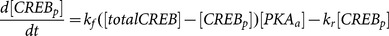
(7)

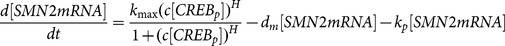
(8)

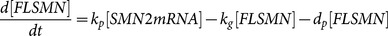
(9)

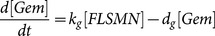
(10)

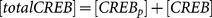
(11)


Both salbutamol and epinephrine directly activate G proteins by binding with their receptors outside the cell. In our model, both compounds were directly substituted for the hormone concentration term in **Equation 1**: 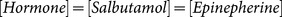
(12)


In addition to activating the *SMN2* promoter, salbutamol also alters the splicing of *SMN2* mRNA causing an increase in *FL-SMN* production over *SMNΔ7* [22]. The interaction of salbutamol with the splicing machinery was modeled as proportional increase in the transcription rate of *SMN2* mRNA given in **Equation 8**:
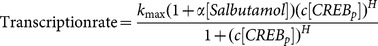
(13)


Treatments with the AC activator forskolin were modeled as an increase in basal AC activity proportional to the dosage of forskolin:
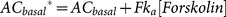
(14)


For the treatments with dbcAMP, the dose concentration was added directly to the existing cAMP concentration in the system:
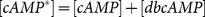
(15)


Rolipram is a well-known PDE inhibitor, which indirectly causes an increase in cAMP. The inhibition interaction was modeled as reversible and competitive in Michaelis-Menten kinetics. The presence of the inhibitor alters the apparent Michaelis constant for the binding of cAMP to PDE:
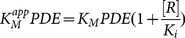
(16)where *[R]* represents the concentration of rolipram and *K_i_* represents the inhibitor dissociation constant.

#### Parameter Estimation

Since the gem count data was collected after a treatment window of 5 days, the concentrations were treated as steady state values for the system. The steady state assumption allowed the model equations to be set to zero and solved for their constituent model states giving a system of algebraic equations. Due to the highly conserved nature of the cAMP pathway, the parameter values derived from the base model were carried over in order to reduce the parameter space to be optimized. A system of equations was derived through the steady state assumption on the full cAMP:*SMN2* model.

The full cAMP:*SMN2* model equations were solved for their principal model states (**Equations 17** **–** **23**). To eliminate the inter-dependence of cAMP and PKA_a_, the cAMP variable was solved to give an equation for cAMP in terms of PKA (**Equation 18**) where Y represents the numerator of the AC term. This equation was then substituted into **Equation 19** and solved for PKA using the equation solver in MATLAB (**S1 Document**). 
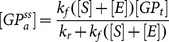
(17)

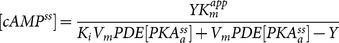
(18)

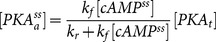
(19)

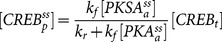
(20)

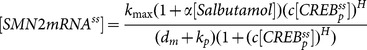
(21)

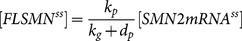
(22)

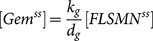
(23)


Parameter estimation was completed through a sign squared error (SSE) analysis between the gem steady state concentrations derived from the gem count data and the full cAMP:*SMN2* model calculations for each compound and dose. The parameter space was covered by generating a multidimensional matrix encompassing the possible combinations of parameter values over a specified range for each parameter to be estimated. For an easier model fit, the two controls were assumed to be identical and their data was averaged together.

### Alternate cAMP:*SMN2* Model

#### Model Development

An alternate pathway has been suggested in the literature [39] which shows active PKA directly stimulating the self-assembly of SMN into gems. Gems are much more stable than monomeric SMN so increasing the gem formation rate would increase the overall SMN concentration over time. Burnett et al. also found no noticeable increase in SMN mRNA levels coinciding with increased PKA_a_ levels suggesting that transcription is not involved. In order to examine the implications of the alternate pathway, a new model was developed by modifying our full cAMP:*SMN2* model.

**Equations 1**, **2** and **3** from the full cAMP:*SMN2* model were also used for this model. The key modifications made were the elimination of the CREB and transcription terms, simplification of translation to a constant rate, T, and the addition of PKA_a_ concentration to the gem formation term. The elimination of the CREB term also allowed for removal of the related conservation equation form the model. The alternate cAMP:*SMN2* model parameters are defined in **Table 3**.
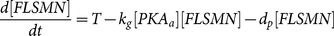
(24)

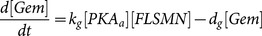
(25)

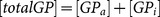
(26)

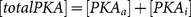
(27)


**Table 2:** Normalized local sensitivity of gem concentration to full cAMP:*SMN2* model parameters.

Parameter	Sensitivity
H	−3.94×10^−3^
GP k_a_	1.54×10^−5^
PKA k_f_	1.36×10^−5^
d_m_	1.31×10^−5^
k_max_	1.22×10^−5^
PKA k_r_	1.19×10^−5^
α	−1.03×10^−5^
k_g_	−7.08×10^−6^
Fk_A_	6.86×10^−6^
GP k_r_	6.70×10^−6^
d_p_	6.50×10^−6^
CREB k_f_	5.98×10^−6^
V_max_PDE	−5.63×10^−6^
c	5.36×10^−6^
PKA k_i_	4.84×10^−6^
CREB k_r_	4.50×10^−6^
AC_basal_	4.36×10^−6^
KI	−2.73×10^−6^
k_p_	2.33×10^−6^
GP k_f_	1.98×10^−6^
d_g_	−1.97×10^−6^
K_m_PDE	−1.53×10^−6^

**Table 3:** Optimized parameter values for cAMP pathway alternate cAMP:*SMN2* model.

Parameter	Description	Value	Units	Source
GP k_f_	G-Protein Activation Rate	1.33×10^−7^	(mMmin)^−1^	This Work
GP k_r_	G-Protein Deactivation Rate	2.05×10^−7^	min^−1^	This Work*
PKA k_f_	PKA Activation Rate	5.37	(mMmin)^−1^	This Work*
PKA k_r_	PKA Deactivation Rate	0.65	min^−1^	This Work*
AC_basal_	Base AC Activation Rate	1.00×10^−5^	mM/min	This Work*
GP k_a_	G-Protein Catalysis Rate	87.83	min^−1^	This Work*
PKA K_I_	PKA Inhibition Rate	1419.09	mM^−1^	This Work*
V_m_	Max cAMP Degradation Rate	9.56	min^−1^	This Work
K_m_	PDE Dissociation Rate Constant	6.66	mM	This Work
T	Transcription Rate	5.42×10^−12^	mM/min	This Work
d_p_	FL-SMN Degradation Rate Constant	2.70×10^−3^	min^−1^	[39]
k_g_	Gem Formation Rate Constant	0.38	min^−1^	This Work
d_g_	Gem Degradation Rate Constant	7.70×10^−4^	min^−1^	[39]
Fk_a_	Forskolin Catalysis Rate Constant	0.05	mM^−1^	This Work
α	Salbutamol Splicing Constant	5149.72	mM^−1^	This Work

The parameters marked with an asterisk (*) were carried over from the simplified cAMP model.

The modifications made for each of the compounds were identical to the full cAMP:*SMN2* model, except that the effect of salbutamol on splicing was represented as a proportional increase in the translation rate (**Equation 28**): 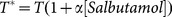
(28)


#### Parameter Estimation

Parameter optimization for the alternate cAMP:*SMN2* model was completed using the steady state SSE analysis described earlier. The G protein, cAMP and PKA steady state equations were carried over from the full cAMP:*SMN2* model and the FL-SMN (**Equation 29**) and gem (**Equation 30**) state equations were derived accordingly. 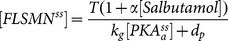
(29)

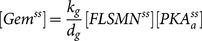
(30)


### Sensitivity Analysis

Steady state sensitivity analysis was completed according to the following equation:
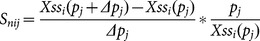
(31)where Sn_ij_ is the normalized sensitivity of species i with respect to parameter j, Xss_i_ is species i at steady state, p_j_ is parameter j, and Δp_j_ is the change in parameter j. The sensitivities of gem concentration and FL-SMN concentration to changes in each of the system parameters were calculated.

### Statistical Analysis

All quantitative data were expressed as mean ± standard error. Comparisons made between quantitative data were made using one-way ANOVA with a Bonferonni *post hoc* test. A p-value less than or equal to 0.05 was considered statistically significant. Statistical analyses of quantitative data were completed using SPSS v.22.

## Results

### Effect of Modulating cAMP Signaling on Gem Localization in SMA Fibroblasts

**Figure 2:**
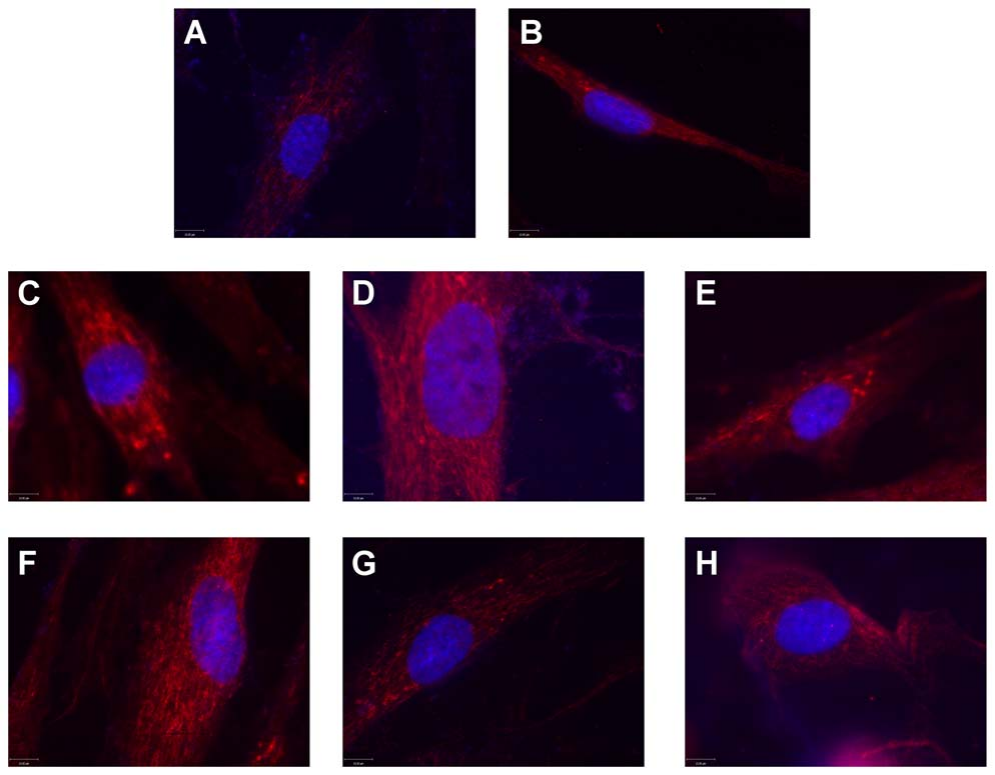
The effect of cAMP signaling modulators on SMN localization to gems. SMN immunostaining (red) of GM03813 SMA fibroblasts treated for 5 days with (**A**) 500 µM dbcAMP, (**B**) ddH_2_O (vehicle for **A**), (**C**) 100 nM epinephrine, (**D**) 50 µM forskolin, (**E**) 100 nM salbutamol, (**F**) 10 µM rolipram or (**G**) DMSO (vehicle for **C-F**). (**H**) SMN immunostaining of GM03814 carrier fibroblasts. The nuclei were counterstained with Hoescht 33342 (blue). Scale bar, 13 µm.

**Figure 3:**
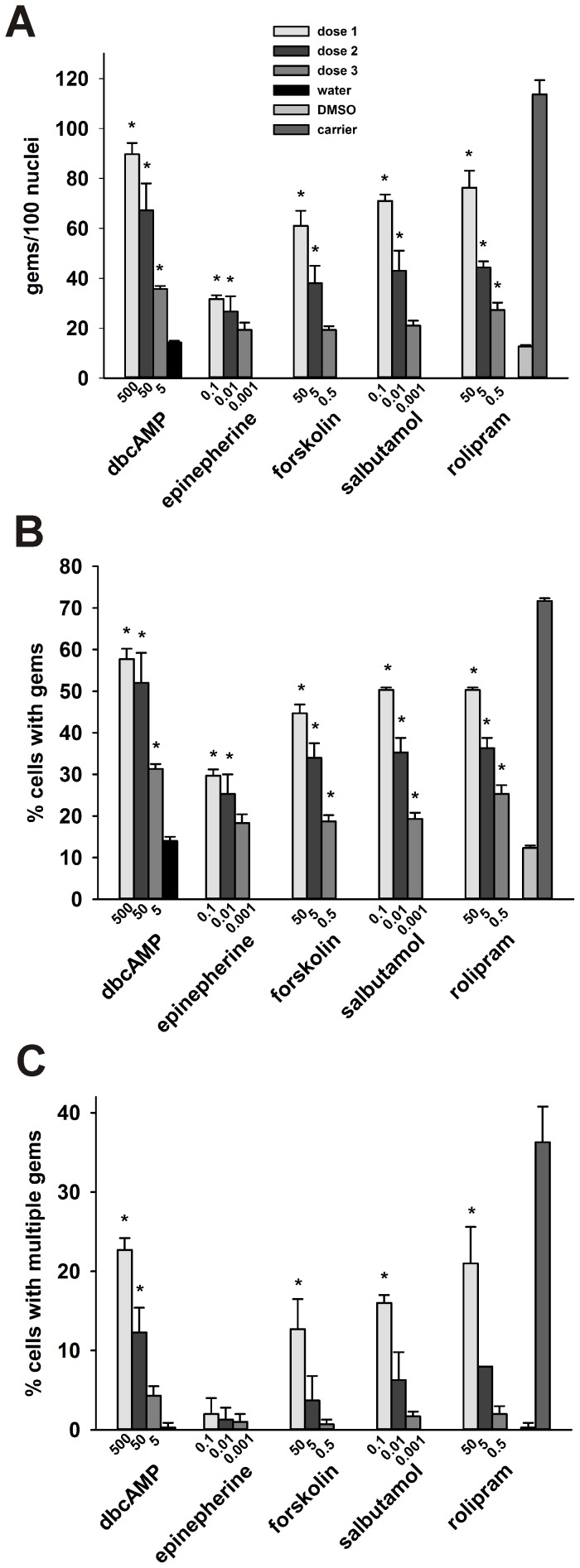
The effects of cAMP signaling modulators on gem counts in SMA fibroblasts. GM03813 SMA fibroblasts were treated with differing doses of dbcAMP, epinephrine, forskolin, salbutamol or rolipram for 5 days (n = 3/dose/drug). The concentrations for each dose of each drug (in µM) are shown below the appropriate bars of each graph. The vehicle for dbcAMP is ddH_2_O (black bars) while DMSO (grey) serves as vehicle for the remaining compounds. The number of SMN-positive nuclear gems was counted in 100 randomly selected nuclei. Gem count analysis was also completed in GM03814 carrier fibroblasts so as to compare the gem data in treated SMA fibroblasts to those observed in healthy cells. The gem count analysis was expressed as (**A**) the number of gems per 100 nuclei, (**B**) the proportion of cells containing gems and (**C**) the proportion of cells containing multiple gems. The asterisk (*) denotes a statistically significant (p≤0.05) difference between drug-treated cells and vehicle (either ddH_2_O for dbcAMP or DMSO for epinephrine, forskolin, salbutamol or rolipram)-treated cells.

Fibroblasts derived from a type II SMA patient (GM03813; [26]) were treated with increasing doses (n = 3/dose) of one of the following modulators of cAMP signaling: epinephrine, salbutamol, forskolin, dbcAMP and rolipram. **Fig. 1** shows how each compound modulates cAMP signaling. All 5 of the cAMP signaling modulators tested increased SMN immunostaining in the nucleus as well as in the cytosol of SMA fibroblasts (**Fig. 2**). Further analysis of SMN localization to gems showed that all 5 of the cAMP signaling modulators increased the number of gems in 100 randomly selected nuclei (**Fig. 3A**), the proportion of cells containing gems (**Fig. 3B**) and proportion of cells containing multiple gems (**Fig. 3C**) in a dose-dependent manner relative to vehicle-treated SMA fibroblasts. While none of the compounds attained gem counts similar to those observed in fibroblasts (GM03814) derived from the mother of GM03813—i.e. carrier fibroblasts, the number of gems/100 nuclei in SMA fibroblasts treated with the highest doses of dbcAMP, forskolin, salbutamol and rolipram reached at least 50% of the gem counts found in carrier fibroblasts.

### Development of a Computational Model for the Effect of cAMP Signaling on *SMN2* Expression

**Figure 4:**
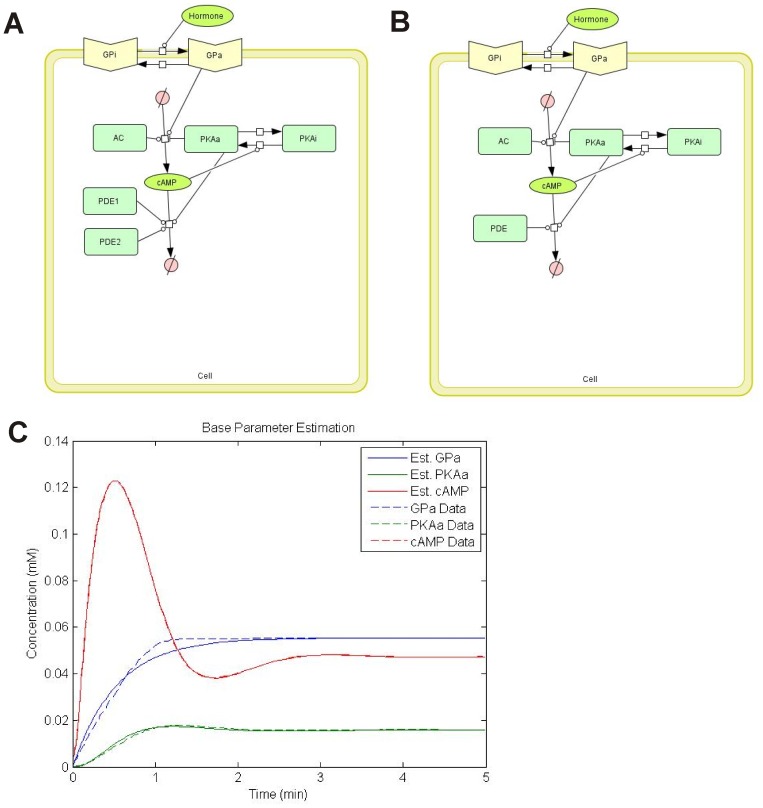
Development of the simplified cAMP:*SMN2* model. Simplified cAMP model captures essential functions of cAMP signaling. (**A**) The original cAMP model generated by Williamson et al. [33]. (**B**) Simplified cAMP model. (**C**) Predicted concentrations of activated G proteins (GPa; blue lines), activated PKA (PKAa; green lines) and cAMP (red lines) following addition of glucose. The solid lines represent the predictions based on the simplified cAMP model while the dashed lines represent data points obtained from [34], [35].

We developed a computational model of cAMP signaling in humans by modifying a previously published model of the cAMP pathway in yeast [33]. Because of the highly conserved nature of cAMP signaling, essential features of the cAMP pathway are common between humans and yeast and can be modeled with limited changes between species. The original model in yeast consists of 3 ordinary differential equations (ODEs) and 16 parameters [33] (**Fig. 4A**). By modeling only those functions essential to cAMP and likely conserved between yeast and humans, our base model of the cAMP pathway contained 3 differential equations and 8 parameters (**Fig. 4B**). Even with this simplified model, we were able to capture the essential dynamics of cAMP signaling reported previously [33] (**Fig. 4C**). Therefore, our computational model represents an essential set of relationships that are likely at work in humans and clinically relevant.

**Figure 5:**
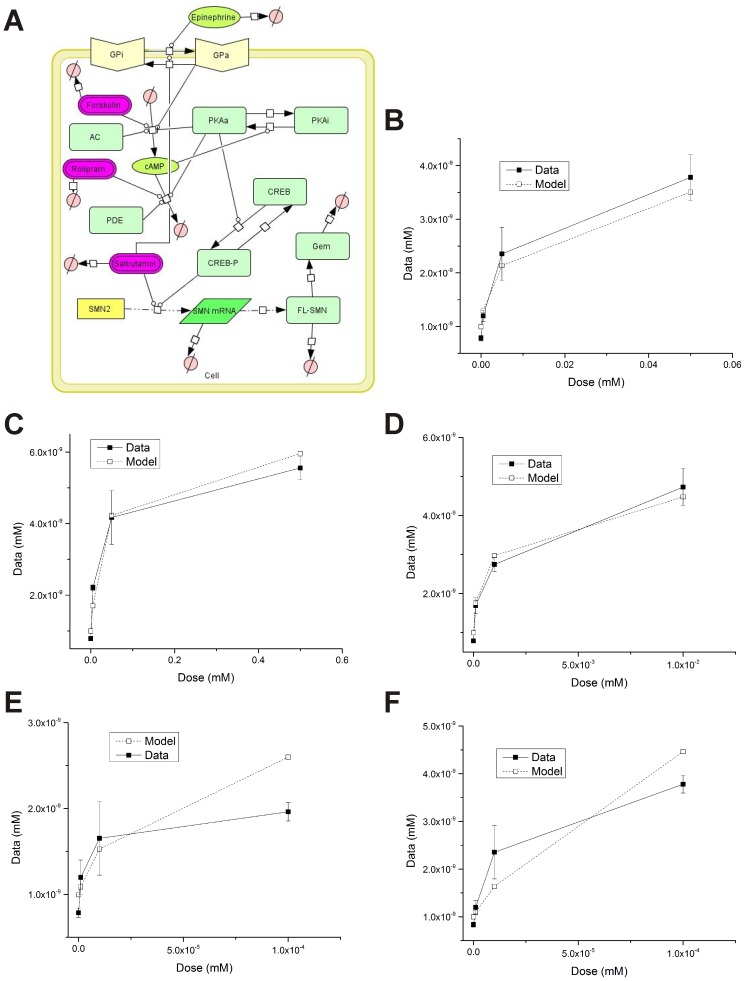
Development of the full cAMP:*SMN2* model. Final gem concentrations of full cAMP:*SMN2* model (**A**) simulations (open squares) compared to experimental data (closed circles) for varying concentrations of forskolin (**B**), dbcAMP (**C**), rolipram (**D**), epinephrine (**E**) and salbutamol (**F**).

We then extended this simplified model of cAMP signaling to include production of FL-SMN through CREB and *SMN2* (**Fig. 5A**). We also added pathways through which treatments to modify cAMP levels may affect FL-SMN production. We used the number of gems/100 nuclei as our experimental dataset—after being converted to concentration (**S1 Table**)—to determine how well our model fits with observed data. Except for those found in the literature, the parameters between cAMP production and gem formation within the computational model were fit to these experimental data by minimizing the sum of squared error (SSE) between simulation and experiment. After preliminary simulation, select parameters from the base equations were optimized to better capture the dynamics observed in the human system (**Table 1**). By comparing model simulations to our experimental data, we can predict which treatments act through the pathways we proposed. The full cAMP:*SMN2* model accurately predicted the response of SMA fibroblasts to treatments with forskolin (**Fig. 5B**), dbcAMP (**Fig. 5C**), and rolipram (**Fig. 5D** **)**. Therefore, it is likely that the overall effects of these drugs act through the cAMP signaling cascade as modeled. Both epinephrine (**Fig. 5E**) and salbutamol (**Fig. 5F**) treatments, however, showed deviations from model predictions. The full cAMP:*SMN2* model predicted a nearly linear, dose-dependent increase in gem formation while the data predicted a sharp increase in gem formation followed by a plateau. The data suggest saturation of the G protein-coupled receptors that bind both epinephrine and salbutamol. These compounds may act through additional, cAMP-independent pathways to give their full effect on gem formation or may have additional levels of regulation that were not simulated.

Having built and tested a computational model of FL-SMN production, we next used a local sensitivity analysis to identify which targets in the cAMP signaling pathway may be most beneficial to optimally increase FL-SMN production and gem formation. Gem concentrations at steady-state were used as output measures to identify the sensitivities of FL-SMN production to perturbations in each model parameter (**Table 2**). The Hill coefficient for the binding of phospho-CREB to the CRE present in the *SMN2* promoter had the most impact on the final gem concentration in the full cAMP:*SMN2* model. Since the Hill coefficient affects the rate of *SMN2* transcription, the sensitivity analysis suggests that the transcription of *SMN2* has the greater impact on cAMP signaling-induced gem formation and functional, FL-SMN expression.

### Using Full cAMP:*SMN2* Mathematical Model to Predict the Effects of Combination Treatments on *SMN2* Expression

**Figure 6:**
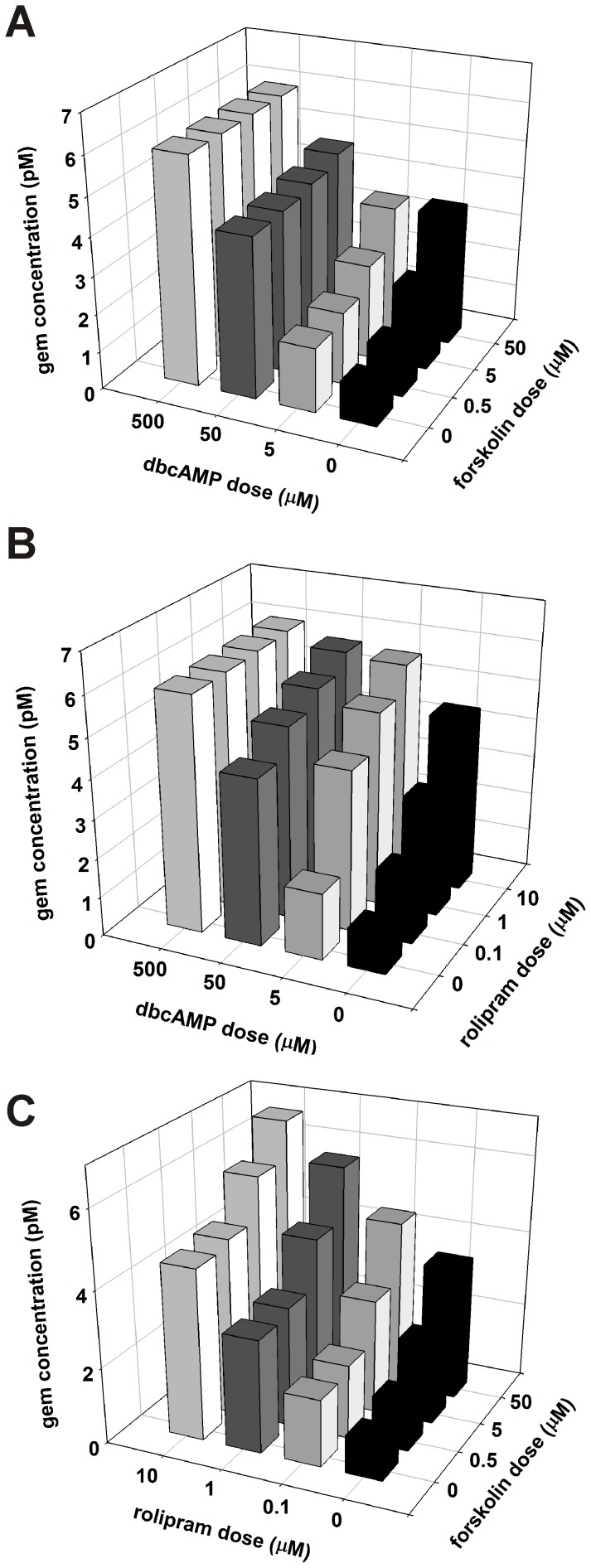
Simulated treatments of SMA fibroblasts with two different cAMP modulators. Dual dose simulation data for the combination treatments with forskolin and dbcAMP (**A**), rolipram and dbcAMP (**B**) and forskolin and rolipram (**C**).

As previously discussed, the full cAMP:*SMN2* model accurately predicts the effects of treatment by rolipram, forskolin and dbcAMP. We used the full cAMP:*SMN2* model to examine the effect of a simultaneous treatment with two of these three compounds: rolipram + dbcAMP, forskolin + dbcAMP and rolipram + forskolin. All three combinations demonstrated synergistic responses yielding higher steady-state gem concentrations with each increasing dose. However, both combinations with dbcAMP hit a maximum gem concentration around 6.0 pM (**Figs. 6A and 6B**) while the combination of rolipram and forskolin peaks around 6.7 pM (**Fig. 6C**). Since the gem concentration in carrier fibroblasts is approximately 7.0 pM (**S1 Table**), these results suggest that manipulation of the cAMP signaling pathway at multiple points in the cascade can optimally increase *SMN2* expression.

### Development of an Alternate Model for the Effect of cAMP Signaling on *SMN2* Expression

**Figure 7:**
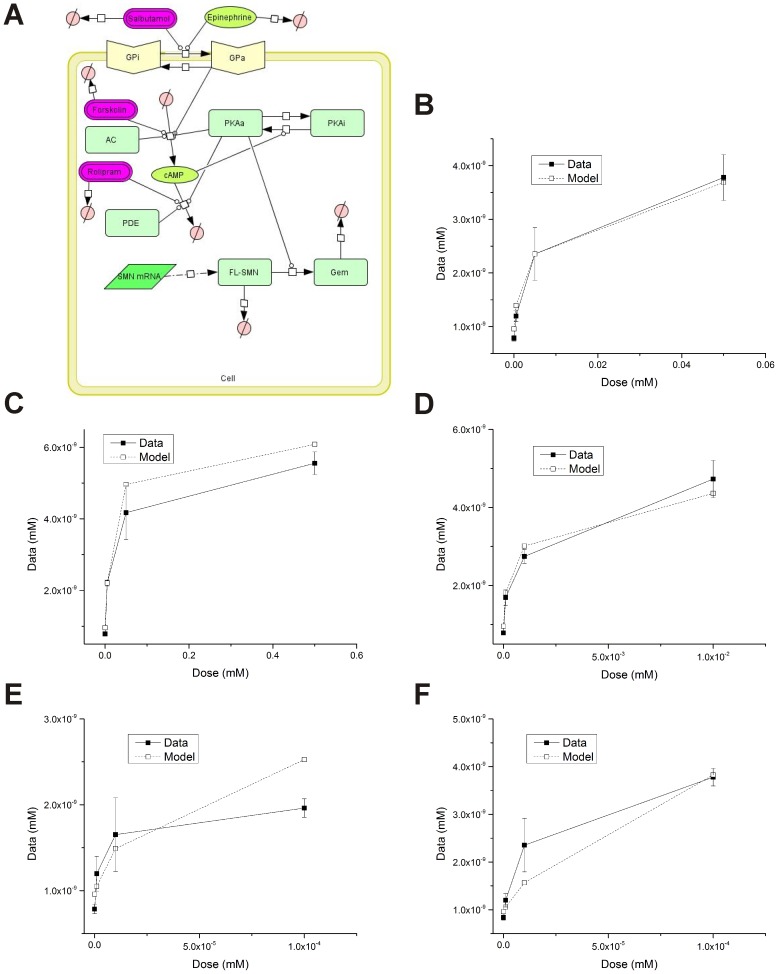
Development of the alternate cAMP:*SMN2* model. Final gem concentrations of alternate cAMP:*SMN2* model (**A**) simulations (open squares) compared to experimental data (closed circles) for varying concentrations of forskolin (**B**), dbcAMP (**C**), rolipram (**D**), epinephrine (**E**) and salbutamol (**F**).

Previous work has shown that activation of PKA directly affects gem formation independent of *SMN2* transcription [39]. Based on this observation, we also developed an alternate model of the interplay between cAMP signaling and the expression of functional FL-SMN and gem formation (the alternate cAMP:*SMN2* model; **Fig. 7A**). We fit the alternate cAMP:*SMN2* model to the available gem concentration data (**S1 Table**) as described for the full cAMP:*SMN2* model. The downstream parameters as well as select simplified model parameters were included for optimization (**Table 3**). Similar to the full cAMP:*SMN2* model, the alternate cAMP:*SMN2* model predicted responses due to treatments with forskolin (**Fig. 7B**), dbcAMP (**Fig. 7C**) and rolipram (**Fig. 7D**); the alternate cAMP:*SMN2* model also yielded deviations for salbutamol (**Fig. 7E**) and epinephrine (**Fig. 7F**) treatments.

With the alternate cAMP:*SMN2* model fully optimized, we conducted a normalized local sensitivity analysis in order to reveal the interactions most important for gem production within the alternate pathway. The alternate cAMP:*SMN2* model sensitivity results show that both the gem formation and degradation rates have a significant influence on final gem concentration, as would be expected (**Table 4**). Furthermore, their sensitivities are three orders of magnitude greater than the corresponding sensitivity to the Hill coefficient in the full cAMP:*SMN2* model. The estimated gem formation rate for the alternate cAMP:*SMN2* model is approximately five orders of magnitude larger than that of the full cAMP:*SMN2* model, thus making this parameter the key differentiator between the two proposed cAMP:*SMN2* models.

**Table 4:** Normalized local sensitivity of gem concentration to alternate cAMP:*SMN2* model parameters.

Parameter	Sensitivity
k_g_	1.00
d_g_	−0.91
K_I_	5.78×10^−4^
PKA k_f_	1.05×10^−4^
PKA k_r_	−1.04×10^−4^
GP k_r_	4.06×10^−7^
T	3.79×10^−7^
GP k_f_	3.18×10^−7^
α	2.29×10^−7^
d_p_	−1.86×10^−7^
GP k_a_	−1.62×10^−7^
FkA	1.37×10^−7^
AC_basal_	−9.77×10^−7^
V_m_PDE	9.24×10^−7^
PKA K_i_	9.05×10^−7^
K_m_PDE	1.33×10^−7^

We also developed and analyzed a combination model which encompasses the effects of cAMP signaling on the upregulation of *SMN2* promoter activity and transcription (the full cAMP:*SMN2* model) as well as the direct stimulation of gem formation by PKA (the alternate cAMP:*SMN2* model). The fit of this combination cAMP:*SMN2* model to the experimental data, however, was significantly weaker than for the fits of either the full cAMP:*SMN2* and alternate cAMP:*SMN2* models (data not shown). Therefore, this combinatorial effect of cAMP signaling on the expression of functional *SMN2* is far less likely to be biologically accurate.

## Discussion

In this study, we use a systems biology approach with mathematical models to characterize the regulation of *SMN2* expression by cAMP signaling. This is the first time, to our knowledge, that a systems biology approach has been used to develop SMA therapeutic strategies. We focused on the interaction between cAMP signaling and *SMN2* expression in this study since there is ample evidence in the literature showing that induction of cAMP signaling increases *SMN2* expression [21]–[23]. The cAMP signaling treatment data were used to generate two distinct mathematical models—full cAMP:*SMN2* and alternate cAMP:*SMN2* models—for the interaction of cAMP signaling and *SMN2* expression. Simulated data from both models match very well with experimental data suggesting that either model is robust. The mathematical models can also be used to predict the effects of drug combinations on cAMP-mediated regulation of *SMN2* expression. This study will also guide future investigations into the mechanisms by which cAMP signaling regulated *SMN2* expression.

Gem formation serves as an indicator of the expression of fully functional, FL-SMN protein in this study. Reduced gem formation correlates with SMN protein expression and disease severity in fibroblasts derived from SMA patients [9]. Numerous studies have identified drug compounds that increase the number of gems in SMA patient cells [27], [40]–[49]. In these studies, drug-induced changes in gem formation are verified by corresponding changes in SMN protein levels in SMA fibroblasts measured by immunoblot or enzyme-linked immunosorbent assays (ELISAs). Some of these gem inducers also increase SMN protein levels in the central nervous system and can ameliorate the phenotype in SMA mouse models [47], [50]–[52]. These observations support the rationale for using gem formation as an indicator of SMN expression in our mathematical models.

Activation of cAMP signaling increases *SMN2* expression but there is debate as to how this signaling cascade regulates *SMN2*. Some groups report that the regulation of *SMN2* promoter activity and mRNA transcription are influenced by cAMP signaling [21]–[23] but others suggest that the regulation of *SMN2* expression by cAMP signaling occurs post-transcriptionally, i.e. by influencing FL-SMN protein stability [39]. The two mathematical models generated in this study for the interaction between cAMP signaling and *SMN2* expression—full cAMP:*SMN2* and alternate cAMP:*SMN2* models—differ by whether or not cAMP signaling affects *SMN2* transcription. Either model fit very well with the experimental data. However, when the two models were combined, the fit with the experimental data was not strong suggesting that only one model correctly simulates the interaction between the cAMP signaling cascade and *SMN2* expression. At present, we cannot determine which mathematical model more accurately simulates cAMP signaling-dependent regulation of *SMN2*. A detailed examination of the time course of gem formation—as well as other levels of *SMN2* regulation—in response to cAMP signaling modulators would permit model discrimination and refinement.

The regulation of SMN expression by cAMP signaling is complex and multi-faceted. As a result, some facets of the interaction between cAMP signaling and SMN gene expression were not included in our mathematical models. For example, PKA has been shown to directly phosphorylate SMN *in vitro* [39], [53]. This PKA-dependent phosphorylation of SMN may modulate its interactions with components of the core SMN:gemins macromolecular complex including gemin-2 (SIP1), gemin-5 and gemin-8 [39], [53]. Since the effects of PKA phosphorylation of SMN on its function and localization are not yet known, we could not factor this variable in our mathematical models. Also, activation of NMDA-type, glutamatergic receptors increases SMN expression in the spinal cord by AKT-mediated phosphorylation of CREB and repression of ERK-activated Elk-1 [54], [55]. It remains to be determined whether cAMP signaling-independent phosphorylation of CREB would affect SMN expression in our model system. Once we have a better understanding of how these events affect SMN expression and function, direct PKA phosphorylation of SMN and the intersection of other signaling pathways—like AKT and MAP kinase (ERK)—can be integrated into mathematical models of the interactions of cAMP signaling on SMN expression and function.

In summary, we have demonstrated that increasing cAMP activation increases FL-SMN production, as measured by gem formation, in human cells using a systems biology approach, We generated data-driven, mathematical models describing the interactions between cAMP signaling and functional FL-SMN production (gem formation) from *SMN2*. These models can be used to identify the components of the cAMP signaling cascade that may be most effective at increasing SMN levels in SMA cells as well as to predict the effects of combination treatment strategies. Development of cAMP signaling-based therapeutic strategies using a combination of biological data and mathematical modeling will be essential for treating SMA.

## Supporting Information

S1 Table Gems concentrations after treatment with cAMP inducing compounds.
(DOCX)

S1 Document Substitution of Equation 18 into Equation 19 to solve for the PKA term.
(DOCX)
