# Supplementary material for: Systems Biology Investigation of cAMP Modulation to Increase SMN Levels for the Treatment of Spinal Muscular Atrophy
Source: PLoS One. 2014 Dec 16;9(12):e115473. doi: 10.1371/journal.pone.0115473 (PMC4267815; doi:10.1371/journal.pone.0115473)
Supplement: S1 Table — Gems concentrations after treatment with cAMP inducing compounds. (DOCX) [file pone.0115473.s001.docx]

| **Compound** | **Dose** | **Gems/100 Nuclei** | **Concentration (pM)** | **SD (pM)** |
| --- | --- | --- | --- | --- |
| forskolin | 50 μM | 61.00 ± 6.00 | 3.78 | 0.377 |
|  | 5 μM | 38.00 ± 7.00 | 2.35 | 0.434 |
|  | 500 nM | 19.33 ± 1.53 | 1.20 | 0.0946 |
| epinephrine | 100 nM | 31.67 ± 1.53 | 1.96 | 0.0946 |
|  | 10 nM | 26.67 ± 6.11 | 1.65 | 0.379 |
|  | 1 nM | 19.33 ± 2.89 | 1.20 | 0.179 |
| dbcAMP | 500 μM | 89.67 ± 4.51 | 5.55 | 0.279 |
|  | 50 μM | 67.33 ± 10.69 | 4.17 | 0.662 |
|  | 5 μM | 35.67 ± 1.15 | 2.21 | 0.0715 |
| salbutamol | 100 nM | 71.00 ± 3.00 | 4.40 | 0.484 |
|  | 10 nM | 43.00 ± 8.00 | 2.66 | 0.0129 |
|  | 1 nM | 21.00 ± 2.00 | 1.30 | 0.323 |
| rolipram | 10 μM | 76.33 ± 6.81 | 4.73 | 0.422 |
|  | 1 μM | 44.33 ± 2.52 | 2.75 | 0.156 |
|  | 100 nM | 27.33 ± 2.89 | 1.69 | 0.179 |
| DMSO | n/a | 12.67 ± 0.58 | 0.785 | 0.0358 |
| ddH_2_O | n/a | 14.33 ± 0.58 | 0.836 | 0.0650 |

**Supplementary Table 1**
