# Supplementary material for: Systems Biology Investigation of cAMP Modulation to Increase SMN Levels for the Treatment of Spinal Muscular Atrophy
Source: PLoS One. 2014 Dec 16;9(12):e115473. doi: 10.1371/journal.pone.0115473 (PMC4267815; doi:10.1371/journal.pone.0115473)
Supplement: S1 Document — Substitution of Equation 18 into Equation 19 to solve for the PKA term. (DOCX) [file pone.0115473.s002.docx]

**Supplementary Document 1**

**Substitution of Equation 18 into Equation 19**

$[PKA_{a}^{ss}]=\frac{Z}{3\left( 2^{\frac{1}{3}} \right)A}-\frac{\left( 2^{\frac{1}{3}} \right)W}{3AZ}-\frac{B}{3A}$

where:

$W=3AC-B^{2}$

$Z=\left( \sqrt{X^{2}+4W^{3}}+X \right)^{\frac{1}{3}}$

$A=PKAk_{r}*V_{m}PDE*PKAK_{I}$

$B=PKAk_{r}{*V}_{m}PDE$

$X=27JA^{2}-9ABC-2B^{3}$

$C = Y(K_{M}^{\mathrm{app}}-\mathrm{PKAk}_{r})$

$J=\left[ totalGP \right]*PKAk_{f}*Y*K_{M}^{app}$

$Y=AC_{basal}+Fk_{a}\left[ Forskolin \right]+GPk_{a}[GP_{a}^{ss}]$
